# Supplementary material for: Identification of photoperiod-induced specific miRNAs in the adrenal glands of Sunite sheep (Ovis aries)
Source: Front Vet Sci. 2022 Jul 22;9:888207. doi: 10.3389/fvets.2022.888207 (PMC9354845; doi:10.3389/fvets.2022.888207)
Supplement: Supplementary file 1 [file Data_Sheet_1.ZIP › Supplementary file/miRDeep2 file.zip!/miRDeep2 file/Know_Structure/LP42A1/oar-mir-382.pdf]

miRBase precursor : oar-mir-382
Total read count : 2870
oar-miR-382-5p read count : 1059
oar-miR-382-3p read count : 1807
remaining reads : 4

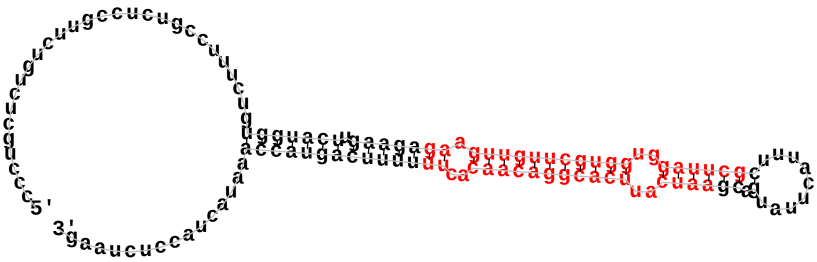

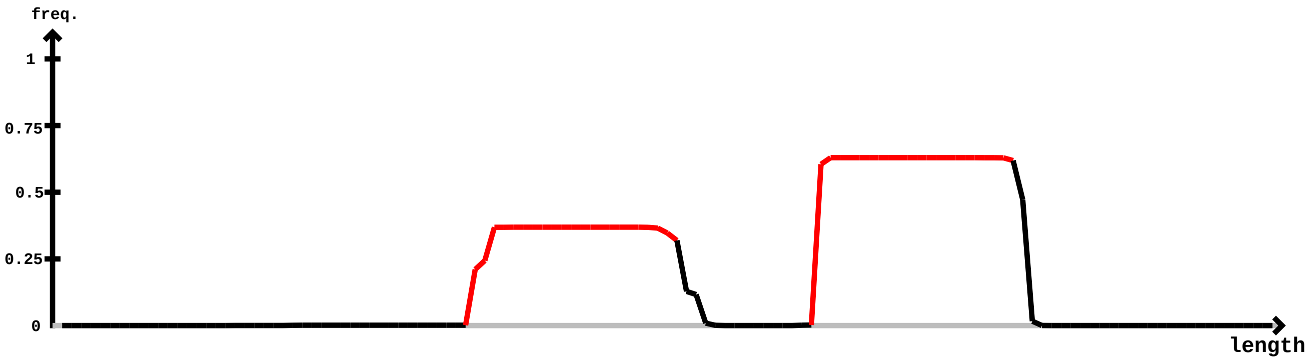


```
                                                                                            oar-miR-382-3p
                                         oar-miR-382-5p
5'- cccugcucugucuugccucugccuuucugugguacuugaagagaaguuguucgugguggauucgcuuuacuuaugacgaaucauucacggacaacacuuuuuucaguaccaaauacuaccucuaag   -3'    exp
    ....((........)).............(((((((.(((((((.(((((((((((..(((((((.........).))))))..)))))))))))..))))))))))))))...............   reads  mm
    .................ucugccuuucugugguacuugaagag...................................................................................   1      0
    .......................uuucugugguacuugaaga....................................................................................   2      0
    ........................uucugugguacuugaaga....................................................................................   1      0
    ..........................................gaaguuguucguggugga..................................................................   2      0
    ..........................................gaaguuguucgugguggau.................................................................   8      0
    ..........................................gaaguuguucgugguggauu................................................................   51     0
    ..........................................gaaguuguucgugguggauuc...............................................................   74     0
    ..........................................gaaguuguucgugguggauucg..............................................................   453    0
    ..........................................gaaguuguucgugguggauucgc.............................................................   15     0
    ...........................................aaguuguucgugguggauu................................................................   2      0
    ...........................................aaguuguucgugguggauuc...............................................................   4      0
    ...........................................aaguuguucgugguggauucg..............................................................   88     0
    ...........................................aaguuguucgugguggauucgc.............................................................   1      0
    ...........................................aaguuguucgugguggauucgcu............................................................   1      0
    ............................................aguuguucgugguggauuc...............................................................   2      0
    ............................................aguuguucgugguggauucg..............................................................   7      0
    ............................................aguuguucgugguggauucgc.............................................................   18     0
    ............................................aguuguucgugguggauucgcu............................................................   306    0
    ............................................aguuguucgugguggauucgcuu...........................................................   23     0
    ............................................aguuguucgugguggauucgcuuu..........................................................   3      0
    ..............................................uuguucgugguggauucgcu............................................................   1      0
    ............................................................................cgaaucauucacggacaacacu............................   2      0
    ............................................................................cgaaucauucacggacaacacuu...........................   2      0
    .............................................................................gaaucauucacggacaacacuu...........................   2      0
    ..............................................................................aaucauucacggacaac...............................   1      0
    ..............................................................................aaucauucacggacaacac.............................   1      0
    ..............................................................................aaucauucacggacaacacu............................   27     0
    ..............................................................................aaucauucacggacaacacuu...........................   414    0
    ..............................................................................aaucauucacggacaacacuuu..........................   1256   0
    ..............................................................................aaucauucacggacaacacuuuu.........................   31     0
    ...............................................................................aucauucacggacaacacuu...........................   3      0
    ...............................................................................aucauucacggacaacacuuu..........................   52     0
    ...............................................................................aucauucacggacaacacuuuu.........................   15     0
    ...............................................................................aucauucacggacaacacuuuuu........................   1      0
```

oar-miR-382-3p

oar-miR-382-5p

cccugcucugucuugccucugccuuucugugguacuugaagagaaguuguucgugguggauucgcuuuacuuaugacgaaucauucacggacaacacuuuuuucaguaccaaauacuaccucuaag
